# Role of Neutrophil-Derived S100B in Acute Myocardial Infarction Patients From the Han Chinese Population

**DOI:** 10.3389/fcvm.2020.595446

**Published:** 2021-03-16

**Authors:** Minghui Cheng, Xu Su, Dan Liu, Xiaoxiang Tian, Chenghui Yan, Xiaolin Zhang, Yaling Han

**Affiliations:** Cardiovascular Research Institute and Department of Cardiology, The General Hospital of Northern Theater Command, Shenyang, China

**Keywords:** S100B, acute myocardial infarction, genotype, thrombosis, plasma biomarkers

## Abstract

**Objective:** This study aimed to clarify the novel role of homeostatic calmodulin S100B and determined whether S100B genetic variants affected atherosclerosis progression in acute myocardial infarction (AMI) patients.

**Methods:** Plasma levels of S100B were measured systemically in AMI patients, stable angina pectoris patients, and control subjects. S100B was obtained from the human coronary artery thrombi using a thrombectomy catheter and quantified via immunohistochemical analysis, qRT-PCR and Western blot analyse. We also screened for S100B variations (rs9722, rs9984765, rs2839356, rs1051169, and rs2186358) via direct sequencing, and investigated the relationship between these variants and AMI patients in the Chinese Han population.

**Results:** Plasma S100B levels increased significantly in AMI patients compared to the levels in stable angina pectoris patients and control subjects (119.45 ± 62.46, 161.96 ± 73.30, and 312.91 ± 127.59 pg/ml, respectively). Immunohistochemical staining results showed that S100B expression was increased in the neutrophils of coronary artery thrombi obtained from AMI patients, as compared to that in normal blood clot, and S100B expression was significantly increased in fresh thrombi tissues, as compared to that in organized thrombi tissues. Western blot and qRT-PCR analysis showed that S100B expression increased in coronary artery thrombi, as compared to that in normal blood clots. After pre-treating the neutrophils with siRAGE, the neutrophils migration induced by S100B were abolished through the NFκB-IL1β/IL6 signaling pathway. Compared to their corresponding wild-type genotypes, the S100B rs9722 variant was associated with increased susceptibility to AMI (OR = 1.35, 95%CI: 1.12–1.65, *P* = 0.02). Individuals with the S100B 9722 A allele had higher plasma S100B levels than those with the G allele in control subjects and AMI patients (141.70 ± 76.69 vs. 107.31 ± 56.05 and 347.13 ± 148.94 vs. 273.05 ± 133.62, respectively).

**Conclusions:** Levels of neutrophil-derived S100B, a novel homeostatic calmodulin, were elevated in the early stages of myocardial infarction. The S100B rs9722 allele was independently associated with AMI patients in the Han Chinese population.

## Highlights

- Levels of neutrophil-derived S100B, a novel homeostatic calmodulin, are elevated in the early stages of myocardial infarction.
- S100B induces neutrophils migration through the NF-κB-IL6/IL1β signal pathway.
- S100B rs9722 allele is independently associated with AMI patients in the Han Chinese population.

## Introduction

Acute myocardial infarction (AMI), the leading cause of death in the industrialized world, is associated with high morbidity and mortality. It is challenged for doctors to rapidly and precisely diagnose AMI and take effective steps for evidence-based medical management and treatment (1, 2). Acute coronary thrombus formation, considered to be secondary to the rupture of vulnerable atherosclerotic plaques, is the main pathological basis for the occurrence of AMI. Atherosclerotic plaque disruption and intramural thrombus formation cause ischemia and hypoxia in cardiomyocytes, which leads to cardiomyocytes necrosis (3–5).

Recent studies have shown that the inflammatory response plays a vital role in the origin and development of atherosclerosis, which causes the rupture of vulnerable atherosclerotic plaques (5–7). During atherosclerotic plaque formation, the knock-on effects caused by inflammatory factors aggravated the degree of atherosclerosis, leading to systemic inflammation and an atherogenesis feedback loop (8, 9). Increasing evidence supports the involvement of circulatory inflammatory cytokines in early vascular inflammation and atherogenesis (10, 11). S100B is composed of at least 25 low-molecular-weight Ca2^+^-binding protein belonging to the S100 family member mainly which is mainly localized in the central nervous system, peripheral nerves, adipocytes and a subset of lymphoid cells under normal physiological conditions. These proteins are involved in various intracellular inflammatory signaling pathways. Interestingly, the S100B bound to the receptor for advanced glycation end products (RAGE) has been shown to play multiple roles in regulating cell functions including proliferation and differentiation, under physiological and pathological conditions. Moreover, S100B also mediates the process of neuroprotection by minimizing and reducing inflammatory factors. Cai et al. (12) demonstrated that S100B participated in RAGE-activated inflammatory pathways and accelerated the onset of coronary artery atherosclerosis. Increased S100B levels were associated with the acute coronary syndrome, and S100B expression was related to myocardial injury in rat models of myocardial infarction. A growing body of evidence suggested that vulnerable plaques in coronary arteries exhibited higher levels of S100B expression which revealed that S100B could play a potential role in atherothrombosis (12).

Therefore, in this study we measured the plasma S100B level and investigated its clinical significance in relation to the occurrence of AMI. Furthermore, we determined whether the genetic variations of S100B were associated with AMI patients in Chinese Han population.

## Materials and Methods

### Study Design and Participants

A total of 367 unrelated Han Chinese subjects including 125 AMI patients, 122 stable angina pectoris patients, and 120 control volunteers were recruited from the General Hospital of Northern Theater Command between August 2015 and October 2017. Information regarding conventional cardiovascular risk factors, such as smoking, hypertension, or diabetes was obtained via a standardized interview. BMI was calculated as the body weight (kg)/height (m)^2^. Type 2 diabetes mellitus was diagnosed according to WHO criteria, and hypertension was diagnosed according to the Seventh Report of the Joint National Committee on the prevention, detection, evaluation, and treatment of high blood pressure, which defined hypertension as arterial blood pressure ≥140 mm Hg (systolic) or 90 mm Hg (diastolic). All recruited subjects with specific clinical presentations and angiographic findings were diagnosed as AMI, stable angina pectoris subjects, and control subjects were recruited from the emergency room. Control subjects were individuals with normal coronary angiogram results without a history of coronary artery disease, electrocardiographic signs of CAD, regional wall motion abnormalities, or relevant valvular abnormalities according to analysis echocardiograms. The design of the present study complied with the Declaration of Helsinki. Approval was obtained from the General Hospital of Northern Theater Command ethics committees, and all subjects provided their informed consent.

### Neutrophils Activation

Neutrophils were isolated via centrifugation density gradient centrifugation. After centrifugation, blood was separated into several distinct bands. Mononuclear cells were placed in the upper phase, whereas the neutrophils became sedimented into the lower phase. Furthermore, we cultured the neutrophils collected from the participants in six well plates (10^6^ cells/mL). Neutrophils were stimulated with 100 ng/mL lipopolysaccharide (LPS) for 4 h in a CO_2_ incubator. Neutrophils not subjected to LPS treatment were used as controls for each participant. At the end of 4 h, the supernatant was separated via centrifugation and stored at −80°C. S100B levels were measured in the cell culture supernatant using a commercial ELISA kit as per the manufacturers' instructions. The LPS-induced secretion of S100B was calculated as the ratio of S100B levels in LPS-stimulated plasma to the corresponding levels in unstimulated plasma obtained from the same patient.

### Human Thrombus Tissue Obtained From the AMI Patients

Human coronary artery thrombi were obtained using a thrombectomy catheter (Export catheter, Medtronic, Minneapolis, MN, USA) during percutaneous coronary intervention prior to balloon angioplasty and stent deployment in AMI patients. The coronary thrombus tissues were obtained by using an aspiration catheter. The expression of S100B and MPO (EPR20257, ABCOM, USA) in the coronary artery thrombus was examined using immunohistochemistry and immunofluorescence staining. S100B and MPO antibodies were incubated overnight at 4°C with thrombus samples; the bound S100B and MPO antibodies were stained using avidin–biotin–peroxidase method (Vector Laboratories, Burlingame, CA, USA) and photographed under a microscope (ZEISS, Image A2, Germany).

### RNA Extraction and qRT-PCR

Fresh thrombus tissues and neutrophils were suspended in 500 μL of TRIzol LS reagent (10296010; Invitrogen, USA) and stored at −80°C prior to RNA isolation. RNA was extracted from the cells in TRIzol, as per the manufacturer's recommended protocol. RNA was quantified using an Eppendorf Biophotometer (Eppendorf, USA). One microgram of RNA was used for cDNA conversion using the Prime ScriptTMRT reagent kit and the gDNA Eraser kit (RR047A, Takara, Japan). Relative levels of S100B expression in the thrombectomies were determined using qRT-PCR (13). The S100B forward primer for qRT-PCR was 5′-GCGAATGTGACTTCCAGGAA-3′ and the reverse primer was 5′-GCTTCCTAATTAGCTACAAC-3′ while the GAPDH forward primer for qRT-PCR was 5′-AGGATGGTGTGGCTCCCTTG-3′ and the reverse primer was 5′-GCAGGGCTGAGACAGCTTCC-3′. Thrombus tissue cDNA was amplified, electrophoresed on 1% agarose gel as described previously, and visualized via ethidium bromide staining. Relative gene expression levels were calculated by comparing the threshold cycle (Ct) for the target gene to that of GAPDH. The mRNA level of S100B in the thrombus tissues of AMI patients and controls was calculated using the following equation: relative gene expression = 2^−(Δ*Ctsample*−Δ*Ctcontrol*)^.

### Protein Extraction and Western Blotting

Neutrophils and thrombus tissues were dissolved in the protein lysis buffer containing a fresh protease inhibitor (14). The cell debris was extracted via centrifugation at 12,000*g* for 10 min at 4°C, and supernatants were extracted for western blotting. The supernatants were electrophoretically separated via SDS-PAGE, and transferred to activated PVDF membranes. PVDF membranes were incubated with the anti-S100B antibody (ab4066, Abcam, USA) and anti-GAPDH antibody (D16H11, Cell Signaling, USA). Immune complexes were detected using the SuperSignal @ West Pico Chemiluminescent Substrate (Thermo Scientific, USA).

### Genetic Variation and Genotyping

A total of 453 AMI patients and 456 control subjects were recruited at the General Hospital of the Northern Theater Command from July 2015 to December 2017.The inclusion criteria for AMI patients were based on a recent AMI definition depending on elevated plasma hscTnT levels (≥0.05 ng/mL) (14), electrocardiogram-related changes such as pathological Q waves, ST segment elevation or depression, and ischemic-type chest pain within the last 24 h. The control participants were selected from subjects who were admitted to the General Hospital of Northern Theater Command who exhibited ≤ 20 % major coronary artery stenosis according to the results of coronary arteriography. Subjects with active inflammatory disease, autoimmune disease, severe heart failure, hemodynamic instability, suspected myocarditis or pericarditis, diseases of the hematopoietic system, extensive of kidney or liver disease, malignant disease, renal or hepatic diseases, taking immune-suppressing drugs, and who had renal or hepatic diseases were excluded.

Genomic DNA was extracted using the DNA extraction kit (TIANGEN, China) according to the manufacturer's instructions (15). Five polymorphisms (rs9722, rs9984765, rs2839356, rs1051169, and rs2186358) of S100B were monitored. A standard polymerase chain reaction (PCR) was used to amplify the S100B rs9722, rs9984765, rs2839356, rs1051169, and rs2186358 variants. Each PCR reaction was performed using 0.1 μg gDNA, 10 pmol each of forward and reverse primer, 0.4 mmol/L dNTPs, 1 × reaction buffer including MgCl_2_ and 4U rTaq DNA polymerase. S100B primer sequences and PCR cycling conditions are included in Table A1.

### Determination of Plasma S100B Levels

Blood samples obtained from AMI, stable angina pectoris patients, and control subjects were dispensed into EDTA-anticoagulation tubes. Plasma samples were isolated and stored at −80°C in plastic cryovials. S100B concentrations were evaluated via ELISA. Each sample used to perform into three experimental replicates and incubated for 2 h. Plates were washed three times, and the conjugate was added and incubated for 2 h at room temperature. Plates were washed again three times, and then incubated for 30 min before the addition of 50 μL of stop solution. Next, we next examined whether the S100B rs9722 GA variant influenced the expression of S100B by measuring the plasma S100B levels between AMI patients and control subjects.

### Transfection of siRNA Into Human Neutrophils

Transient siRNA transfection was conducted in the light of the advice provide by the manufactory. We transfected the neutrophils with siRNA in a transfection medium using the liposomal transfection reagent (Lipofectamine RNAimax, Invitrogen) and the neutrophils grown in 6 well plates were transfected. In the 150 μl transfection medium added 6 μl siRNA solution and 150 μl transfection medium added 6 μl transfection reagent, thus gently mixed together. At room temperature for 30 m, we added the siRNA-lipid complexes into the neutrophils with the 1.0 ml transfection medium, and incubated the cells with this mixture for 6 h at 37°C. Furthermore normal medium replaced the transfection medium and neutrophils were cultured for 48 h.

### Transwell Migration Assay

Neutrophils with or without the S100B protein, and neutrophils transfected with the siRNA RGAE medium were added to the transwell migration assay using the 8 μm pore polycarbonate membrane inserts (Corning). Moreover, adding 600 μl culture with 10% fetal calf serum in the lower chamber, and adding 105 neutrophils with 0.1% fetal calf serum in the upper chamber were incubated for 24 h. Then the neutrophils migrating to the lower surface of the membrane were fixed with methanol and stained with crystal violet. The migration neutrophils numbers were calculated by 4 fields per membrane under a microscope.

### Statistical Analysis

Statistical analysis was performed using SPSS version 21.0 and data were represented as mean ± SEM values. We used the Student's *t* test or Mann-Whitney *U* test to compare groups; the plasma S100B levels were analyzed using ANOVA. The distribution of S100B genotype and allele frequencies between AMI and control groups were compared using X^2^ tests. The AMI risk was evaluated by *P*-values, 95% confidence intervals (95% CIs), and odds ratios (ORs). Adjustment were made for bonferroni multiple test comparisons of the five SNPs, and a significance cutoff value of *p* < 0.01 (0.05/5 = 0.01) was applied. Assuming a dominant genetic model, the study had 80% statistical power at the 5% significance level for detecting the effect of the polymorphism and was associated with an OR > 1.8, which indicated a low risk of obtaining a false-negative result.

## Results

### Increased S100B Plasma Levels in AMI Patients

**Figure 1:**
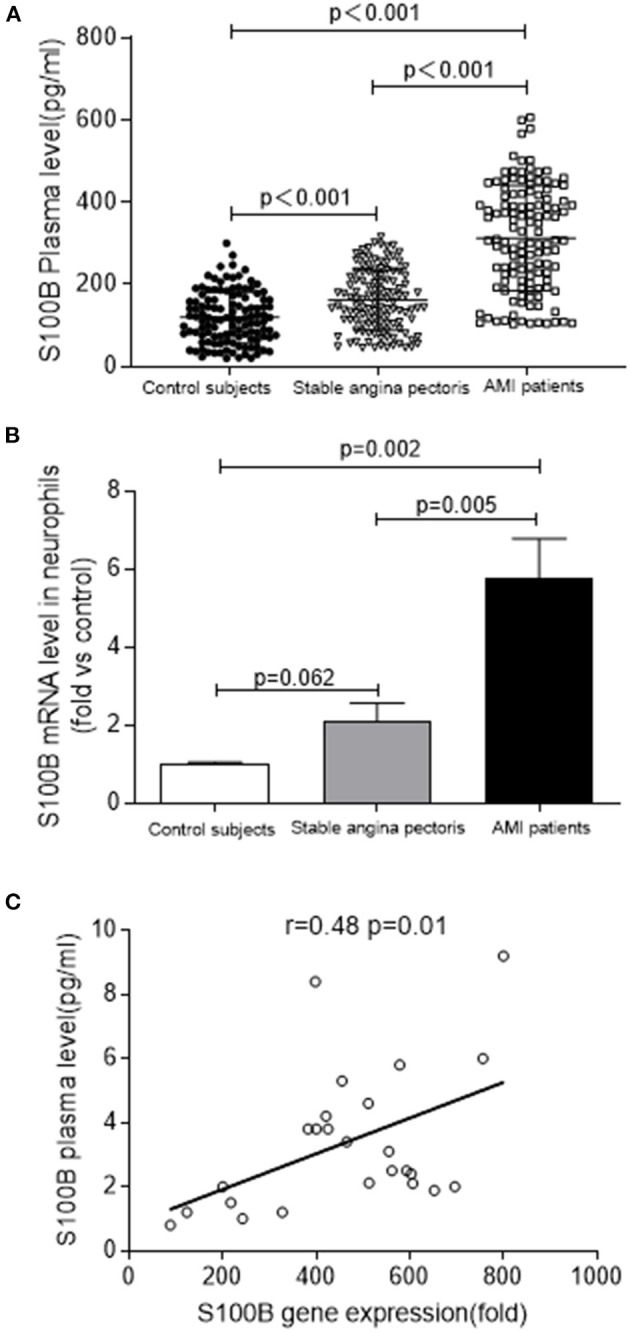
S100B levels are increased in patients with AMI. **(A)** S100B plasma levels in AMI (*n* = 125) and stable angina pectoris (*n* = 122), as compared to those in control subjects (*n* = 120). **(B)** S100B gene expression in the neutrophils of AMI patients (*n* = 10) and stable angina pectoris patients (*n* = 10) as compared to those in control subjects (*n* = 10). **(C)** Correlation between S100B gene levels in the neutrophils and plasma S100B of AMI patients.

We enrolled 367 participants including 120 control subjects, 122 stable angina pectoris and 125 AMI patients. Baseline characteristics and the results of plasma biochemistry tests were presented in Table 1. In comparison to the control subjects, there were more smokers (*P* = 0.007), diabetics (*P* < 0.01), and hypertension patients in AMI patients. The AMI group also had more comorbid conditions than the stable angina pectoris or control groups, and the patients were more likely to have a history of stroke (*P* = 0.022). As expected, the clinical and biochemical values of AMI patients were significantly higher than those of subjects belonging to the control and stable angina pectoris groups. Clinical parameters including higher WBC levels, which indicate poorer cardiac functioning and general conditions, and severe inflammation were more prevalent in AMI group than in the control or stable angina pectoris groups. AMI patients (*n* = 125) had significantly higher systemic S100B levels, as compared to those in stable angina pectoris (*n* = 122) and controls subjects (*n* = 120) [median (IQR):302.16 (606.50–103.18) vs. 159.50 (315.69–45.38) vs. 119.45 (300.23–20.41)] (Figure 1A).

**Table 1:** Baseline demographic and clinical parameters among the healthy control, stable angina pectoris and AMI subjects.

**Variable**	**Healthy Controls** **(*n* = 120)**	**Stable angina pectoris patients** ** (*n* = 122)**	**AMI patients (*n* = 125)**	***P*-value**
Age, years	58.68 ± 11.44	60.09 ± 11.97	60.88 ± 13.27	0.238
Female, no. (%)	42 (35.0)	47 (38.5)	47 (37.6)	0.841
Smoking	30 (25.0)	53 (43.4)	50 (40.0)	0.007
Hypertention	44 (36.7)	61 (50.0)	88 (70.4)	0.000
Diabetes	13 (10.8)	51 (41.8)	71 (56.8)	0.000
Previous stroke	0 (0.00)	7 (5.70)	8 (6.4)	0.022
Previous AMI	0 (0.00)	5 (4.10)	8 (6.40)	0.023
Family history	3 (2.50)	3 (2.50)	8 (7.20)	0.178
WBC, ×10^9^/L	5.97 ± 1.55	7.02 ± 1.90	13.30 ± 3.53	0.000
TG, mmol/dl	1.45 ± 0.54	1.63 ± 0.92	1.61 ± 0.81	0.216
LDL-C, mmol/dl	2.28 ± 0.66	2.30 ± 0.79	2.80 ± 0.71	0.007
hscTnT, ng/mL	0.008 ± 0.004	0.011 ± 0.012	4.53 ± 3.52	0.000
CK-MB, U/L	11.98 ± 6.51	15.69 ± 5.72	245.76 ± 235.21	0.000

*TG, triglyceride; LDL-C, low-density lipoprotein; WBC, white blood cell; hs-CRP, high-sensitivity C-reactive protein; hscTnT, high-sensitivity troponin T; CK-MB, creatine kinase MB isoenzyme*.

To confirm the fact S100B was highly upregulated in AMI patients, we analyzed S100B expression in neutrophils using real time PCR in a cohort of patients with AMI. We observed the marked upregulation of S100B in AMI patients (*n* = 10), as compared to that control subjects (*n* = 10) and stable angina pectoris patients (*n* = 10) (*P* = 0.062; *P* = 0.005; *P* = 0.002) (Figure 1B). However, the S100B level was also upregulated in stable angina pectoris patients as compared to the level in control subjects, albeit at a lower level than in AMI patients. Finally, we observed that S100B mRNA expression and plasma levels were positively correlated in AMI patients, which suggested that neutrophils might act as an important source of S100B in AMI patients (*r* = 0.48, *P* = 0.01) (Figure 1C). In AMI patients, neutrophils released S100B into the plasma, resulting in a marked increase in plasma S100B levels during the early stages of AMI.

**Figure 2:**
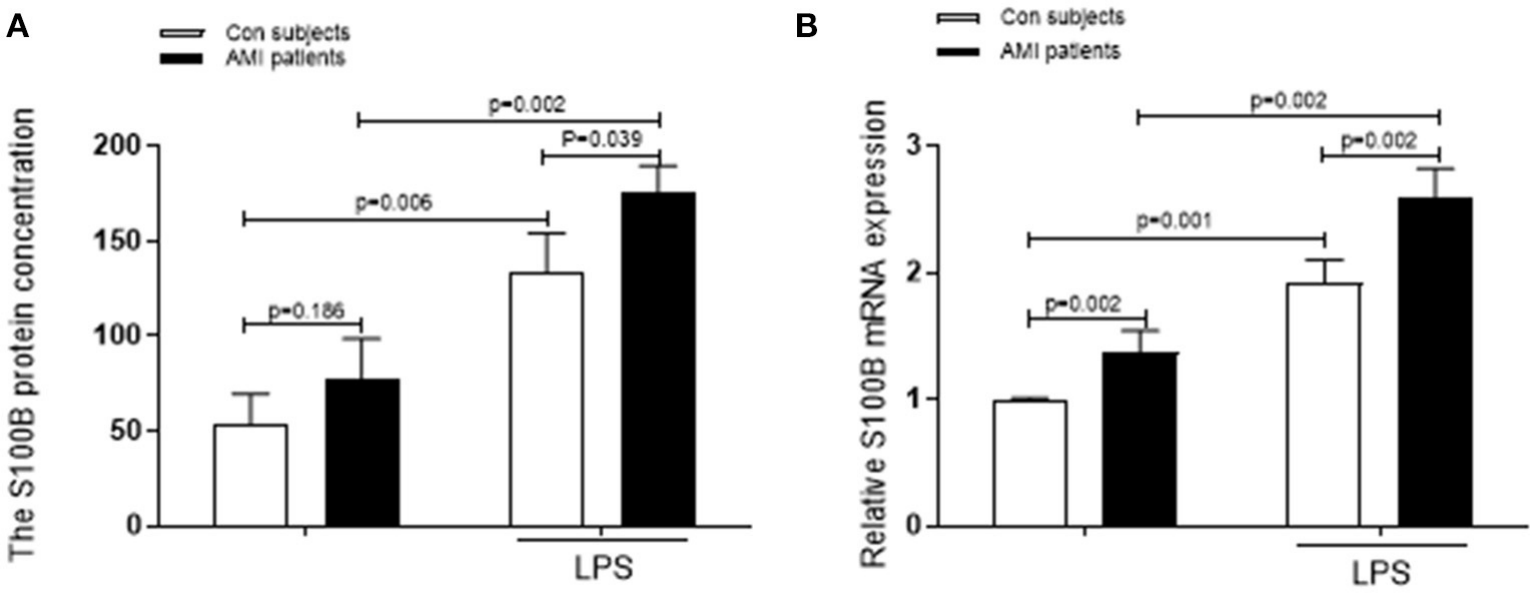
Levels of S100B secreted by stimulated neutrophils are increased in AMI patients. **(A)** A comparision of the fold change in the levels secreted by LPS-stimulated neutrophils with that in unstimulated neutrophils from the same AMI patients (*n* = 10) and control subjects (*n* = 10). **(B)** The S100B mRNA levels and the corresponding secreted S100B levels in the unstimulated controls and LPS-stimulated subjects are shown.

We also evaluated the differences in the levels of pro-inflammatory S100B secreted after activation in AMI patients and control subjects. We measured S100B levels in the supernatants of neutrophils stimulated with LPS for 4 h. In the control and AMI subjects, secreted levels of S100B increased in LPS stimulated-neutrophils, as compared to those in unstimulated neutrophils (*p* = 0.006; *p* = 0.002). In neutrophils isolated fromAMI patients, S100B protein and mRNA expression levels increased similarly after LPS stimulation, with significantly different levels of S100B being expressed from the control-stimulated neutrophil supernatant (*p* = 0.001; *p* = 0.002). S100B levels were increased in LPS-stimulated cells, as compared to those in both controls and AMI subjects (Figure 2).

### S100B Expression Increased in the Thrombi Tissues Obtained From AMI Patients

**Figure 3:**
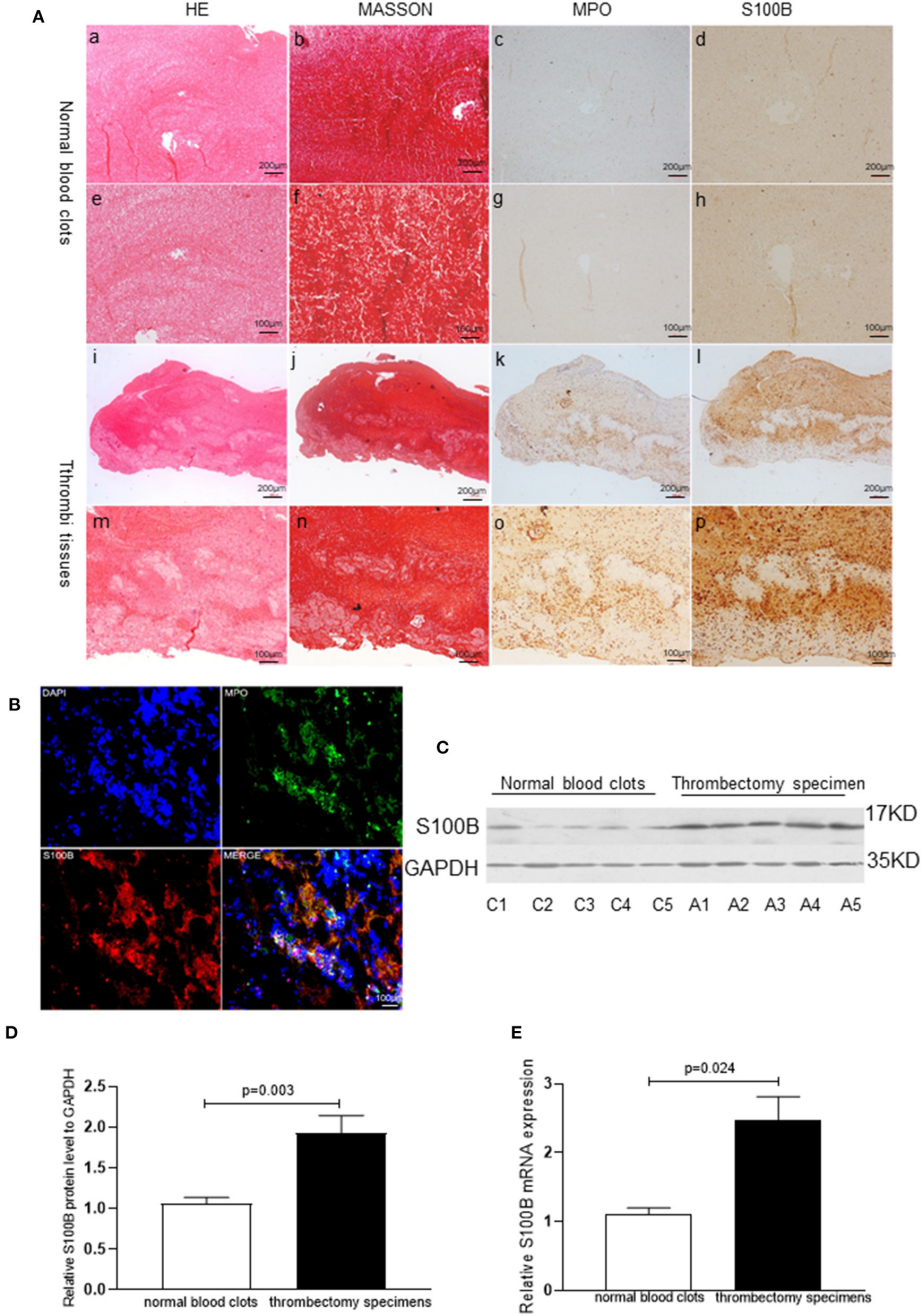
S100B expression in human thrombi tissues of AMI patients. **(A)** Representative images showing the immunohistochemical staining of MPO and S100B in thrombi tissues coming obtained from AMI patients. Normal blood clots: a, b (×10) and e, f (×20): Thrombi tissues: i, j (×10) and m, n (×20). S100B expression was observed in thrombi tissues: d, h, i, p; MPO^+^ neutrophils were identified in thrombi tissues (c, g, k, o) and S100B expression was increased in areas with high levels of neutrophils infiltration. **(B)** Representative immunofluorescence staining results of MPO and S100B from human thrombi tissues. S100B proteins were co-localized in the neutrophils of thrombi tissues. **(C,D)** Western blot analysis of S100B expression in normal blood clots and thrombectomy specimens obtained from AMI patients. S100B protein was detected in all specimens, but was present at significantly higher levels in the thrombectomy specimens (*n* = 5) as compared to levels in normal blood clots (*n* = 5). C1–C5 showed representative normal blood clots from control subject 1 control subject 5. A1–A5 show the representative images of thrombectomy specimens from AMI patient 1 to patient 5. **(E)** qRT-PCR comparison of S100B mRNA expression levels of normal blood clots and thrombectomy specimens obtained from AMI patients. Image magnification ×10 and ×20.Immunoreactivity was detected using DAB staining (brown color).

**Figure 4:**
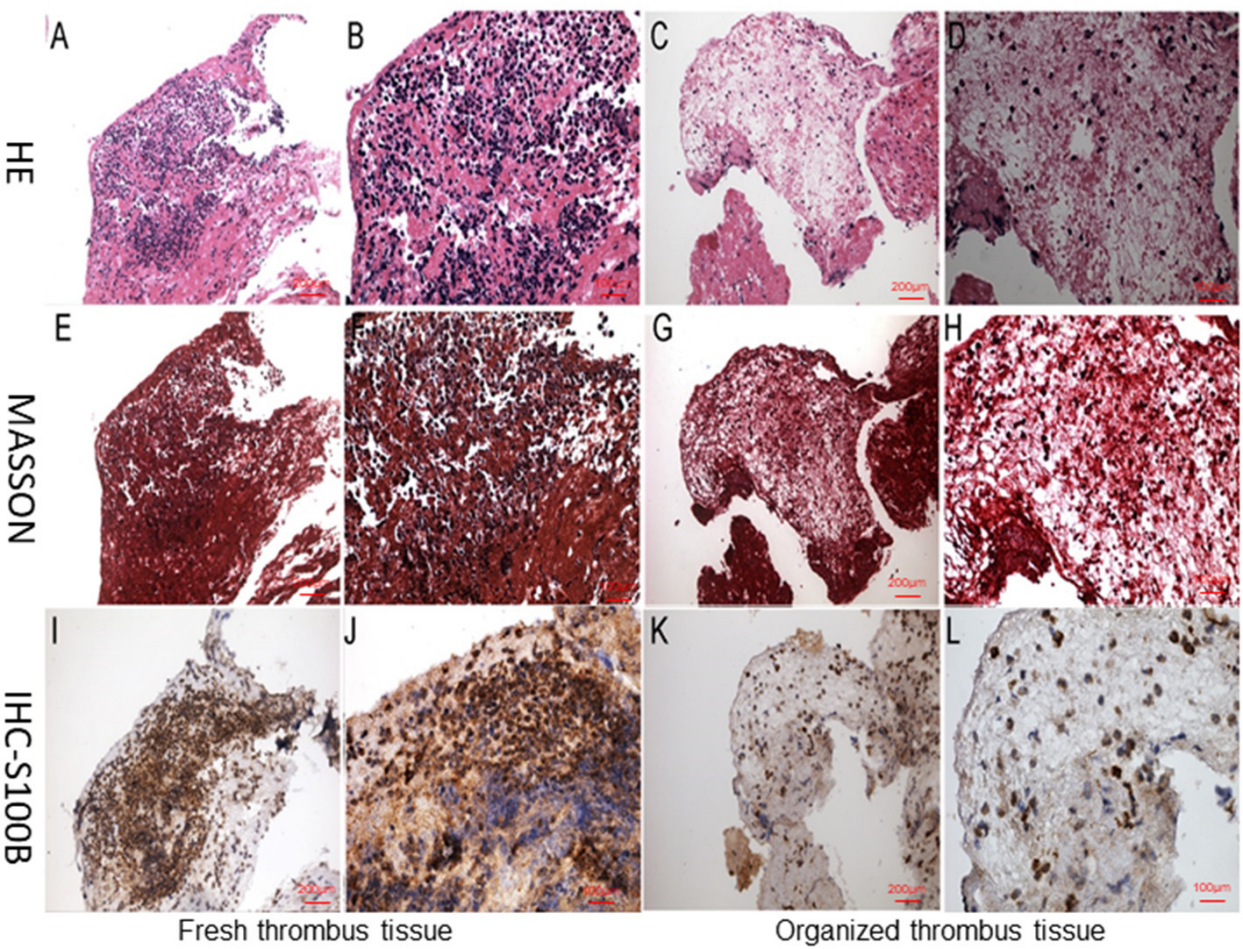
Neutrophil staining during different stages of thrombus organization. **(A,B,E,F,I,J)** Fresh thrombus tissue with numerous visible neutrophils. **(C,D,G,H,K,J)** Organized thrombus tissues with largely absent. **(A–D)** H&E staining of thrombus tissues, **(E–H)** Masson staining of thrombus tissues, **(I–L)** Immunohistological staining of thrombus tissues. All samples were histologically graded as either fresh thrombi containing numerous neutrophils, or organized thrombi with a few neutrophils. Image magnification ×10 and ×20.Immunoreactivity was detected using DAB staining (brown color).

Acute coronary thrombus formation, considered to be secondary to the rupture of vulnerable atherosclerotic plaques, leads to the occurrence of AMI. To examine the S100B expression pattern in thrombus tissues, we collected coronary artery thrombi from AMI patients who underwent angiography, and demonstrated the thrombotic occlusion of the anterior descending branch of the coronary artery. Thereafter, we removed thrombus tissues via an aspiration thrombectomy followed by balloon angioplasty and stent deployment; as a result, a widely patent anterior descending branch of the coronary artery exhibited no significant luminal narrowing. The thrombectomy catheter retrieved multiple coronary artery thrombi that were then analyzed for MPO positive neutrophils and S100B expression using immunohistochemical microscopy. MPO and S100B were abundantly expressed and co-localized in the thrombus tissue. S100B expression was significantly increased in positive neutrophils from the coronary artery thrombus of AMI patients as compared to that in normal blood clots. Numerous neutrophils were visible, in fresh thrombus tissues, but were largely absent in organized thrombus tissues. The H&E staining and Masson staining of fresh thrombus tissues showed that the S100B expression of neutrophils was higher than that in fresh thrombi (Figures 3, 4).

S100B expression levels in human thrombus tissues were further analyzed in five blood clots and five thrombectomy specimens using qRT-PCR (*P* = 0.024) and western blotting (*P* = 0.003). S100B expression levels increased in the thrombi, as compared to those in the normal blood clot tissues, which indicates that S100B might play an important role in AMI progression (Figure 3).

### Association Between S100B Variants and Occurrence of AMI Patients

To avoid false positive experimental results, the DNA source, preparation, and genotyping analysis were controlled using the paradigm of blindness and randomization. The demographic data for 453 AMI patients and 456 control subjects is presented in Table 2. We screened for S100B variations (rs9722, rs9984765, rs2839356, rs1051169, and rs2186358) via direct sequencing and investigated the relationship between these variants and AMI in the Han Chinese population. The distribution frequency of genotypes conformed to the Hardy-Weinberg equilibrium in the control subjects. AMI patients were characterized by higher frequencies of traditional atherosclerotic risk factors, compared to the control subjects. Cardiovascular risk factors including hypertension, diabetes mellitus, and cigarette smoking were more prevalent in AMI patients than in control subjects. Furthermore, lower levels of HDL-C and increased levels of TG were found in the AMI patients as compared to those observed in the control subjects.

**Table 2:** Clinical characteristics of AMI patients and control subjects.

**Variable**	**Control subjects**	**AMI patients**	***P*-value**
	**(*n* = 456)**	**(*n* = 453)**	
Female, no. (%)	146 (32.0)	155 (34.0)	match
Age (years)	58.97 ± 12.12	60.83 ± 10.33	0.001
BMI	25.48 ± 3.03	25.76 ± 2.94	0.459
Smoking	247 (24.2)	274 (60.5)	<0.001
Hypertension	265 (28.1)	266 (58.7)	<0.001
Diabetes	137 (30.0)	176 (38.9)	0.003
TG, mmol/dl	1.29 ± 0.53	1.78 ± 0.88	<0.001
TC, mmol/dl	3.97 ± 0.63	4.21 ± 0.54	0.012
LDL-C, mmol/dl	2.26 ± 0.72	3.12 ± 0.61	0.022
HDL-C, mmol/dl	1.01 ± 0.33	1.04 ± 0.21	<0.001

*BMI, body mass index; F, female; M, male; HDL-C, high density lipoprotein cholesterol; LDL-C, low density lipoprotein chlolsterol; TC, total cholesterol; TG, triglyceride*.

The differences in the allelic and genotypic frequencies of the S100B variants rs9722, rs2239574, rs881827, rs9984765, and rs1051169 were compared between AMI patients and control subjects for S100B in the Han Chinese population. Carriers of the rs9722 AA allele were significantly overrepresented among AMI patients as compared to those of control subjects (15.0% vs. 7.9%, *P* = 0.002). S100B rs9722 is independently associated with AMI patients in the Han Chinese population (*P* = 0.002, AA vs. GG; OR = 1.35,95%CI: 1.12–1.65, *P* = 0.002) (Table 3). After adjusting for classical cardiovascular risk factors such as age, gender, body mass index, smoking, hypercholesterolemia, hypertension and diabetes, the association between AMI patients and S100B rs9722 remained significant. There were no significant differences in the allele frequencies or genotype distributions of the other four SNPs (rs2239574, rs881827, rs9984765, and rs1051169) between AMI patients and control subjects. When the subjects were categorized into sub-groups according to gender, the AA genotype and A allele frequencies of rs9722 were found to be significantly higher in male patients than in male controls (*P* = 0.006; OR = 1.39;95% CI = 1.09–1.78, Table 4). The AA genotype and A allele frequency of rs9722 between female patients and female controls was not higher (Table 4).

**Table 3:** Genotypic and allelic frequencies of S100B polymorphisms between the 456 control subjects and 453 AMI subjects.

	**Control subjects**	**AMI patients**	***P***	**OR(95% CI)**
	**(*n* = 456)**	**(*n* = 453)**		
Rs9722				
G/G no. (%)	221 (48.5)	191 (42.2)	0.002	
G/A no. (%)	199 (43.6)	194 (42.8)		
A/A no. (%)	36 (7.9)	68 (15.0)		
G allele	641 (70.3)	576 (63.6)	0.002	1.35 (1.12–1.65)
A allele	271 (29.7)	330 (36.4)		
Rs9984765				
T/T no. (%)	218 (47.8)	205 (45.3)	0.19	
C/T no. (%)	197 (43.2)	203 (44.8)		
C/C no. (%)	41 (9.0)	45 (9.9)		
C allele	279 (30.6)	279 (32.3)	0.42	1.08 (0.88–1.32)
T allele	613 (69.4)	633 (67.7)		
Rs2839356				
G/G no. (%)	226 (40.6)	221 (46.6)	0.14	
C/G no. (%)	199 (43.6)	195 (43.0)		
C/C no. (%)	31 (16.8)	47 (10.4)		
G allele	651 (71.4)	617 (68.1)	0.12	1.16 (0.95–1.42)
C allele	261 (28.6)	289 (31.9)		
Rs1051169				
A/A no. (%)	185 (40.6)	183 (40.4)	0.99	
A/C no. (%)	198 (43.4)	196 (43.3)		
C/C no. (%)	73 (16.0)	74 (16.3)		
A allele	562 (62.0)	568 (62.3)	0.91	1.01 (0.83–1.22)
C allele	344 (38.0)	344 (37.7)		
rs2186358				
A/A no. (%)	363 (79.6)	381 (84.1)	0.20	
A/C no. (%)	84 (18.4)	69 (14.6)		
C/C no. (%)	9 (0.02)	6 (0.01)		
A allele	810 (88.8)	828 (91.4)	0.06	1.33 (0.98–1.82)
C allele	102 (11.2)	78 (8.6)		

OR, Odd Ratio; CI, Confidence Interval. ^*^ P<0.01.

**Table 4:** Genotypic and allelic frequencies of rs8193037 in AMI patients and control subjects according to gender.

**SNP**	**Genotype**	**Females**		***P*-value**	**OR (95%CI)**	**Males**		***P*-value**	**OR (95%CI)**
		**Controls** ** subjects**	**AMI** ** patients**			**Controls** ** subjects**	**AMI** ** patients**		
		**146**	**155**			**310**	**298**		
Rs9722	GG	72 (49.3)	65 (41.9)	0.05		151 (48.7)	124 (41.6)	0.009 ^*^	
	GA	64 (43.8)	66 (42.6)			135 (43.5)	128 (43.0)		
	AA	10 (6.8)	24 (15.5)			24 (7.7)	46 (15.4)		
	G	208 (71.2)	196 (63.2)	0.04	1.44 (1.02–2.03)	437 (70.5)	376 (63.1)	0.006^*^	1.39 (1.09–1.78)
	A	84 (28.8)	114 (36.8)			183 (29.5)	220 (36.9)		

P-values are obtained for the comparison between the AMI and control subjects by χ2 -test;

* *P < 0.01*.

We performed a case-control study of plasma S100B levels to explore the association between S100B expression and AMI.S100B levels were significantly higher in AMI patients as compared to those in the control group. Further analysis revealed that the disease-susceptible genotype S100B rs9722 AA was associated with higher S100B levels in the plasma as compared to the GG and GA genotypes in both controls and AMI patients (141.70 ± 76.69 vs.107.31 ± 56.05 and 23 347.13 ± 148.94 vs 273.05 ± 133.62 respectively) (Table 5).

**Table 5:** Plasma S100B levels in subgroup subjects according to S100B rs9722 G/A genotypes.

**Group**	**Plasma S100B levels (pg/mL)**	
	**GG + GA**	**AA**
Control subjects (*n* = 75)	107.31 ± 56.05	141.70 ± 76.69
AMI patients (*n* = 75)	273.05 ± 133.62^*^	347.13 ± 148.94^*^^$^

*Results are means ± S.D*.

* P < 0.05 compared with GG + GA;

$ *P < 0.01 compared with AA control group*.

### S100B Influences the Effects of Neutrophils Through the RAGE–NFκB–IL1β/IL6 Signaling Pathway

**Figure 5:**
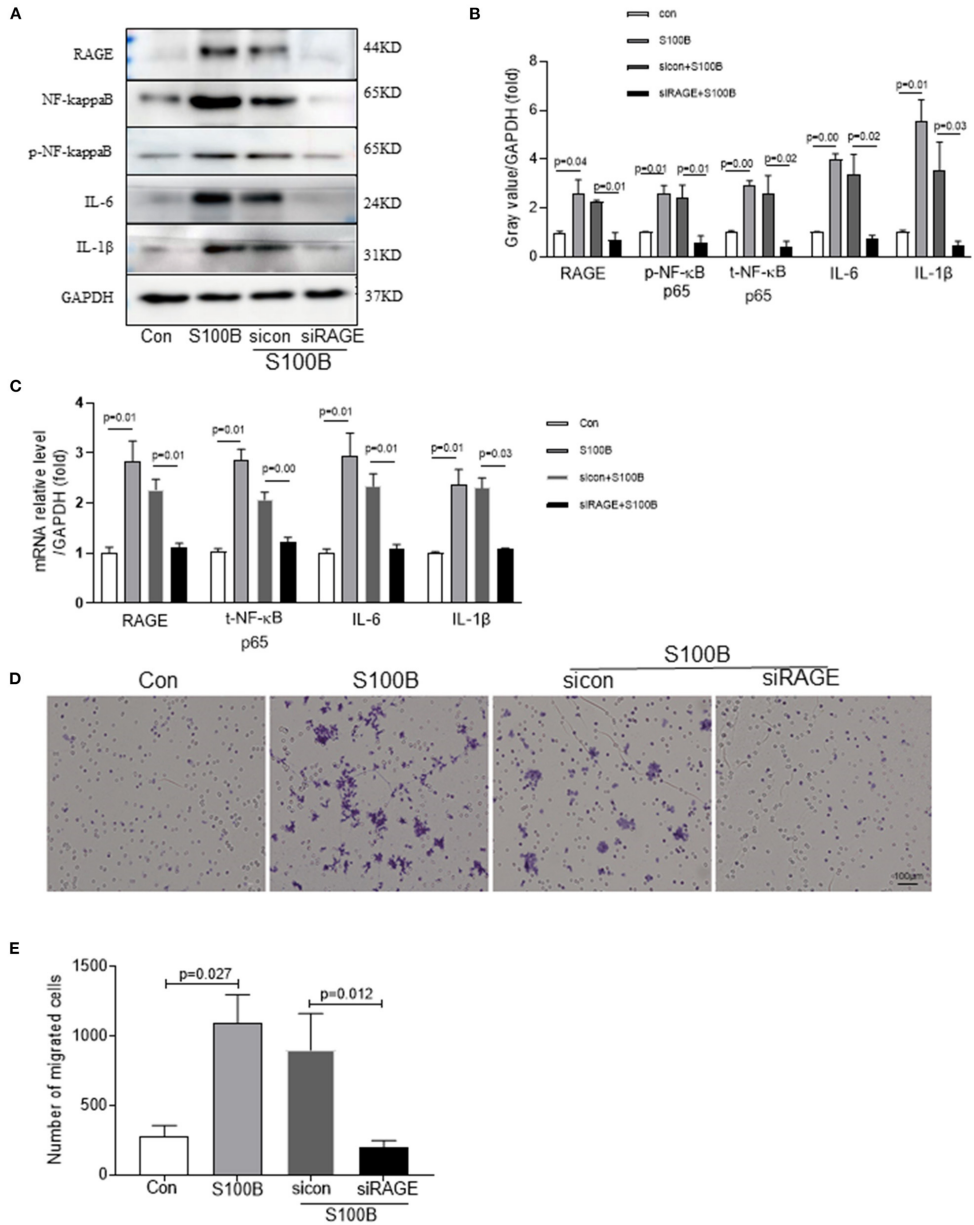
Effect of S100B and S100B receptor knockdown influencing on neutrophils migration through the RAGE–NFκB–IL1β/IL6 signaling pathway. **(A,B)** Western blot analysis of RAGE–NFκB–IL1β/IL6 expression in neutrophils with S100B protein, sicontrol and siRAGE (S100B receptor). **(C)** Quantitative real-time PCR analysis of mRNA level of RAGE–NFκB–IL1β/IL6 in neutrophils with S100B, sicontrol and siRAGE. **(E)** Untransfected or transfected neutrophils with a control siRNA vector or siRAGE to enable the overexpression of the S100B fusion protein, control siRNA, or siRAGE were subjected to the transwell chamber migration assay. Cells that had migrated to the lower surface of the transwell chamber were stained with crystal violet and counted under a microscope. Representative microscopic images are shown. The column chart shows mean numbers of migrated cells in three experiments.

S100B stimulated inflammation and S100B receptor(siRAGE)may confer some cardiovascular benefits by modulating the inflammatory process in atherosclerosis. Therefore, we examined the ability of siRAGE to modulate effect of S100B in neutrophils. To investigate whether S100B induced the inflammatory response in neutrophils which were stimulated with S100B for 2 h. siRAGE induced a significant decrease in RAGE–NFκB–IL1β/IL6 production in S100B-activated neutrophils (Figures 5A–C). To investigate the possible effects of S100B on neutrophils migration, we tested whether the addition of S100B fusion protein or knocking down of S100B in neutrophils would alter their migratory ability. To examine whether S100B induction increased neutrophil migration, we performed neutrophil migration assays under static conditions. S100B treatment significantly altered the migration (Figures 5D,E) of neutrophils. Furthermore, we transfected cultured neutrophils with either the S100B fusion protein/sicontrol and siRAGE, respectively. In separate experiments, cultured neutrophils were transfected with either S100B siRNA or control siRNA. Efficient S100B protein fusion or knockdown has been shown in Figure 5. Transfected cells were subjected to migration assays by using the transwell chamber. The experiments showed that the S100B fusion protein significantly increased the migratory ability of neutrophils whereas RAGE (S100B receptor) knockdown inhibited neutrophils migration (Figure 5).

## Discussion

Coronary heart disease, and its main complication AMI, represented major causes of morbidity and long-term disability worldwide. The exact etiology and pathophysiological mechanisms of AMI have remained unclear till date. The formation of thrombi during the progression of vulnerable plaques is an important factor contributing to AMI development. The cascade amplification effect is thought to promote the progression of atherosclerosis and cardiovascular complications, leading to the occurrence of AMI (2–4).

S100B is small molecular calmodulin that adhered to the RAGE receptor and induces the inflammatory reaction of the coronary artery unstable plaque, thus, it aggravated the rupture of unstable coronary artery plaque (13, 16, 17). In the present study, we found that plasma S100B levels were significantly higher in AMI patients than in the control subjects and stable angina pectoris patients. Moreover, we found that the S100B levels in the neutrophils obtained from AMI patients were higher with LPS stimulation compared to neutrophils from control subjects. We also found that organized thrombi from the coronary artery had lower levels of S100B than fresh thrombi from the same AMI patients. Furthermore, we found that S100B induced the neutrophil migration through the NF-KB-IL6/IL1β signal pathway; a blockade of this pathway interrupted neutrophil migration. Taken together, our results suggested that S100B might play a crucial role in the occurrence of AMI, especially in the early stages of myocardial infarction.

The current study verified the association between S100B rs9722 and the risk of AMI in the Han Chinese population. After adjusting the various risk factors, the S100B rs9722 variant was found to be associated with AMI patients. We demonstrated that AMI patients with the homozygous rs9722 AA allele exhibited increased levels of plasma S100B in AMI patients. Moreover, we found that rs9722 was located in 3′-UTR region of S100B, and the variants of S100B could alternate the potential microRNA targeting sites in S100B. Using the TargetScan version 4.0 (http://www.targetscan.org/), it was predicted that the S100B rs9722 A allele disrupted the binding sites of miRNAs (hsa-miR-3912-3p, hsa-miR-340-3p, hsa-miR-6797-3p), which could influence the expression of S100B (18–20). A minimum “seed” length of 7-mer was specified as the minimum cut-off score. It was possible that the S100B rs9722 variant at the 3′-UTR affected gene expression at the post-transcriptional level by interfering with protein binding, polyadenylation, or microRNA (miRNA) binding, which would further influence S100B expression.

The S100B expressed by the neutrophils in the coronary artery of AMI patients was examined, the results suggested that S100B were released from the shoulder region of ruptured coronary artery plaques of the AMI patients. Thus, S100B might be released into the blood immediately from the inflammatory cells in the shoulder region of ruptured coronary artery plaques, leading to a rapid increase in plasma S100B levels. Moreover, we found that organized thrombi had lower levels of S100B than fresh thrombi tissue obtained from the AMI patients which suggested that S100B may be released from the thrombi tissues into the blood during the early stages of AMI; thus, plasma S100B levels could be significantly higher at the onset of myocardial infarction.

There are several limitations associated with the present study (14, 15). As observed in numerous previous studies, the validity results are highly dependent on the study sample size. To verify these results, the study cohort needs to be expanded to include a larger number of participants, as well as a more racially heterogenous cohort. Second, the functional mechanisms underlying the association between the S100B rs9722 polymorphisms and AMI risk require further investigation, which can be achieved using high-throughput “omics” technology. By expanding the cohort across different geographical regions, the relationship between the S100B rs9722 variant and AMI risk could be further clarified, and the underlying mechanisms may be revealed. Most importantly, the elevation of S100B levels might not be AMI specific, and might represent a non-specific response to an acute body injury; it could be a secondary inflammatory response observed after the occurrence of AMI. Not only is the S100B level increased in patients with cardiovascular disease but it is also increased in those with non-cardiovascular disease. Further experiments are needed to investigate the findings of present study.

In summary, the level of novel homeostatic calmodulin in S100B is elevated in the early stages of myocardial infarction, and the S100B variant rs9722 is independently associated with AMI patients in the Han Chinese population.

## Data Availability Statement

The raw data supporting the conclusions of this article will be made available by the authors, without undue reservation.

## Ethics Statement

The studies involving human participants were reviewed and approved by the General Hospital of Northern Theater Command ethics committees. The patients/participants provided their written informed consent to participate in this study. Written informed consent was obtained from the individual(s) for the publication of any potentially identifiable images or data included in this article.

## Author Contributions

YH was responsible for the study concept and design and obtained study funding. YH, MC, and XZ wrote the manuscript. XS, DL, XT, and CY collected clinical specimens. MC and XZ did the statistical analysis. All authors critically revised the paper and approved the final version.

## Conflict of Interest

The authors declare that the research was conducted in the absence of any commercial or financial relationships that could be construed as a potential conflict of interest.

## Appendix

**Table A1:** Primer Sequence used in genotyping *S100B* variations.

**SNPs**	**Primer sequence**	**Size of PCR product (bp)**	**Annealing temperature**
Rs9722	forward:5′-AAGAGCAGGAGGTTGTGGAC-3′	291	58°C
	reverse:5′- AAGAGGTTTTCAATTTTTCA -3′		
Rs9984765	forward:5′- GATCCAAGGA CAGTGGAGAC-3′	421	55°C
	reverse:5′- GGCTTTAGCTA GAAGTTTAG-3′		
Rs2139356	forward:5′- GGGACCTTGA TGCACCTACT -3′	371	56°C
	reverse:5′- CTGAGCTGGA GAAGGCCATG-3′		
Rs1051169	forward:5′- TTGAGGTCTG TATTGATAAC-3′	451	52°C
	reverse:5′- GAGTAACGGAACCCTCAGTG -3′		
Rs2186358	forward:5′- CAGATGGATT TTAAGAGGAG -3′	531	62°C
	reverse:5′- ATCCCTGAAT TCCCTTGCTG-3′		

## References

[1] Osmak GJ Titov BV Matveeva NA Bashinskaya VV Shakhnovich RM Sukhinina TS. Impact of 9p21.3 region and atherosclerosis-related genes' variants on long-term recurrent hard cardiac events after a myocardial infarction. Gene. (2018) 647:283–8. 10.1016/j.gene.2018.01.036 29331485

[2] Tahto E Jadric R Pojskic L Kicic E. Neutrophil-to-lymphocyte ratio and its relation with markers of inflammation and myocardial necrosis in patients with acute coronary syndrome. Med Arch. (2017) 71:312–5. 10.5455/medarh.2017.71.312-315 29284896 PMC5723203

[3] Hansson GK. Inflammation, atherosclerosis, and coronary artery disease. N Engl J Med. (2005) 352:1685–95. 10.1056/NEJMra043430 15843671

[4] Koenen RR Weber C. Chemokines: established and novel targets in atherosclerosis. EMBO Mol Med. (2011) 3:713–25. 10.1002/emmm.201100183 22038924 PMC3377113

[5] Garcia de Tena J. Inflammation, atherosclerosis, and coronary artery disease. N Engl J Med. (2005) 353:429–30. 10.1056/NEJM200507283530425 16049220

[6] Mehta JL Saldeen TG Rand K. Interactive role of infection, inflammation and traditional risk factors in atherosclerosis and coronary artery disease. J Am Coll Cardiol. (1998) 31:1217–25. 10.1016/S0735-1097(98)00093-X 9581711

[7] Chuang C-T Guh J-Y Lu C-Y Wang Y-T Chen H-C Chuang L-Y. Steap4 attenuates high glucose and S100B-induced effects in mesangial cells. J Cell Mol Med. (2015) 19:1234–44. 10.1111/jcmm.12472 25817898 PMC4459839

[8] Sentürk T Çavun S Avci B Yermezler A Serdar Z Savci V. Effective inhibition of cardiomyocyte apoptosis through the combination of trimetazidine and N-acetylcysteine in a rat model of myocardial ischemia and reperfusion injury. Atherosclerosis. (2014) 237:760–6. 10.1016/j.atherosclerosis.2014.10.091 25463117

[9] Daffu G del Pozo CH O'Shea KM Ananthakrishnan R Ramasamy R Schmidt AM. Radical roles for RAGE in the pathogenesis of oxidative stress in cardiovascular diseases and beyond. Int J Mol Sci. (2013) 14:19891–910. 10.3390/ijms141019891 24084731 PMC3821592

[10] Monden M Koyama H Otsuka Y Morioka T Mori K Shoji T. Receptor for advanced glycation end products regulates adipocyte hypertrophy and insulin sensitivity in mice: involvement of Toll-like receptor 2. Diabetes. (2013) 62:478–89. 10.2337/db11-1116 23011593 PMC3554382

[11] Okuda LS Castilho G Rocco DDFM Nakandakare ER Catanozi S Passarelli M. Advanced glycated albumin impairs HDL anti-inflammatory activity and primes macrophages for inflammatory response that reduces reverse cholesterol transport. Biochim Biophys Acta. (2012) 1821:1485–92. 10.1016/j.bbalip.2012.08.011 22940078

[12] Cai XY Lu L Wang YN Jin C Zhang RY Zhang Q. Association of increased S100B, S100A6 and S100P in serum levels with acute coronary syndrome and also with the severity of myocardial infarction in cardiac tissue of rat models with ischemia-reperfusion injury. Atherosclerosis. (2011) 217:536–42. 10.1016/j.atherosclerosis.2011.05.023 21663912

[13] Liu D Zhang X-L Yan C-H Li Y Tian X-X Zhu N. MicroRNA-495 regulates the proliferation and apoptosis of human umbilical vein endothelial cells by targeting chemokine CCL2. Thromb Res. (2015) 135:146–54. 10.1016/j.thromres.2014.10.027 25466836

[14] Zhang W Sun K Zhen Y Wang D Wang Y Chen J. VEGF receptor-2 variants are associated with susceptibility to stroke and recurrence. Stroke. (2009) 40:2720–6. 10.1161/STROKEAHA.109.554394 19520980

[15] Wang Y Zheng Y Zhang W Yu H Lou K Zhang Y. Polymorphisms of KDR gene are associated with coronary heart disease. J Am Coll Cardiol. (2007) 50:760–7. 10.1016/j.jacc.2007.04.074 17707181

[16] Meng L Park J Cai Q Lanting L Reddy MA Natarajan R. Diabetic conditions promote binding of monocytes to vascular smooth muscle cells and their subsequent differentiation. Am J Physiol Heart Circ Physiol. (2010) 298:H736–45. 10.1152/ajpheart.00935.2009 20008269 PMC2838549

[17] Ehlermann P Eggers K Bierhaus A Most P Weichenhan D Greten J. Increased proinflammatory endothelial response to S100A8/A9 after preactivation through advanced glycation end products. Cardiovasc Diabetol. (2006) 30:5–6. 10.1186/1475-2840-5-6 16573830 PMC1475836

[18] Mazaheri M Karimian M Behjati M Raygan F Colagar AH. Association analysis of rs1049255 and rs4673 transitions in p22phox gene with coronary artery disease: a case-control study and a computational analysis. Ir J Med Sci. (2017) 186:921–8. 10.1007/s11845-017-1601-4 28474233

[19] Morini E Rizzacasa B Pucci S Polidoro C Ferrè F Caporossi D. The human rs1050286 polymorphism alters LOX-1 expression through modifying miR-24 binding. J Cell Mol Med. (2016) 20:181–7. 10.1111/jcmm.12716 26542080 PMC4717858

[20] Huang XC Wang W. Association of MEF2A gene 3'UTR mutations with coronary artery disease. Genet Mol Res. (2015) 14:11073–8. 10.4238/2015.September.21.20 26400337

